# A Game of Snakes and Ladders: An Audit of the Management of Patients Presenting With Functional Somatic Symptoms at a Paediatric Tertiary Care Centre

**DOI:** 10.1192/bjo.2025.10530

**Published:** 2025-06-20

**Authors:** Abigail Turley, Amy Jaiteh, Louisa Draper

**Affiliations:** Alder Hey Children’s NHS Foundation Trust, Liverpool, United Kingdom

## Abstract

**Aims:** This audit aimed to evaluate the initial assessment, investigation, management, and communication strategies for paediatric patients presenting with Functional Somatic Symptoms at Alder Hey Children’s Hospital. The audit addressed a significant gap in existing national NHS and local guidelines for guidance of medical professionals in diagnosing and managing Functional Somatic Symptoms presenting to paediatric services.

**Methods:** A parallel-running retrospective analysis was performed jointly by the General Paediatrics team and the Child Health Psychology (Liaison) team on young people admitted under General Paediatrics and/or attending outpatient Psychiatric services over a four-month period. Summary narratives were generated for each patient detailing the journey from first presentation with functional symptoms to diagnosis and management. This was then interpreted by systematic data analysis – both qualitative and quantitative.

**Results:** The audit’s key finding was the absence of a standardized approach to the assessment, investigation, and management of Functional Somatic Symptoms. Patients underwent excessive investigations across multiple departments without coordinated multidisciplinary team (MDT) involvement. Furthermore, very few received a formal diagnosis of Functional Somatic Symptoms by discharge; communication and psycho-education around this diagnosis with young people and their families was distinctly lacking.

**Conclusion:** These findings highlight an urgent need for the development of both national and local structured guidelines and integrated care pathways to improve the management of Functional Somatic Symptoms in children and young people across physical and mental health services. This is needed to ensure timely diagnosis, psycho-education, appropriate intervention, and better long-term outcomes for affected young people and their families.

